# Comprehensive analysis of potential biomarkers for the diagnosis and prognosis of Cervical squamous cell carcinoma - based on GEO and TCGA databases

**DOI:** 10.3389/fonc.2025.1524225

**Published:** 2025-05-08

**Authors:** Yufen Chen, Qinghua Deng, Tengyue Fu, Yuxiang Huang, Houlin Li, Jingmu Xie, Feng Liao, Feimiao Zeng, Xinyi Fang, Ruiman Li, Zhuming Chen

**Affiliations:** ^1^ Department of Obstetrics and Gynecology, The First Affiliate Hospital of Jinan University, Guangzhou, Guangdong, China; ^2^ Department of Gynaecology, The Second Affiliated Hospital of Guangdong Medical University, Zhanjiang, Guangdong, China; ^3^ Department of Neurosurgery, The First Affiliated Hospital of Shantou University Medical College, Shantou, Guangdong, China; ^4^ Guangdong-Hong Kong-Macau Institute of CNS Regeneration (GHMICR), Jinan University, Guangzhou, Guangdong, China; ^5^ Reproductive Medical Center, Affiliated Hospital of Guangdong Medical University, Zhanjiang, Guangdong, China

**Keywords:** cervical squamous cell carcinoma (CESC), diagnosis, survival prognosis, immune infiltration, biomarker

## Abstract

**Background:**

Cervical squamous cell carcinoma (CESC) constitutes a substantial global health burden, especially in resource-limited regions. The identification of reliable biomarkers is critical for developing a clinically applicable nomogram to predict survival outcomes and evaluate immune infiltration in CESC patients.

**Methods:**

This study integrated RNA-seq data from GEO and TCGA databases to identify key genes associated with CESC through differential expression analysis and machine learning techniques. Prognostic models were constructed and validated, with additional analyses exploring immune cell infiltration and gene function via GSEA and clinical correlation. Finally, key genes were validated via qRT-PCR in CESC tissues.

**Results:**

A total of 112 differentially expressed genes (DEGs) were identified through differential analysis of the GEO and TCGA datasets. *EFNA1*, *CXCL8*, and *PPP1R14A* emerged as prognostic biomarkers for CESC, showing significant associations with survival, tumor stage, and immune infiltration. *EFNA1* may drive tumor progression via the MAPK signaling pathway, *CXCL8* could influence immune evasion through NOD-like receptor signaling, and *PPP1R14A* may contribute to tumor invasion by modulating extracellular matrix remodeling. A nomogram integrating these genes demonstrated high predictive accuracy for overall survival (AUC>0.75) and calibration plots. Decision curve analysis (DCA) was performed to assess the nomogram’s clinical utility and net benefit for application in clinical practice. Additionally, it was validated by qRT-PCR, showing elevated expression in tumors versus normal tissues (*P*<0.05).

**Conclusion:**

*EFNA1*, *CXCL8*, and *PPP1R14A* are promising biomarkers for CESC prognosis and immune regulation. The nomogram model provides a practical tool for personalized survival prediction, enhancing clinical decision-making for immunotherapy and risk stratification.

## 1 Introduction

CESC is the fourth most common cause of cancer death among women globally, accounting for approximately 606,000 new cases and 342,000 deaths annually (1). Notably, over 70% of cervical cancer deaths occur in low- and middle-income countries (LMICs), where the incidence and mortality rates are the second highest (1, 2). Despite advancements in prevention and treatment, cervical cancer remains a significant health threat to women in LMICs, highlighting disparities in access to care (2). Current treatment strategies for CESC depend on the diagnostic stage. For stage I, hysterectomy is the primary treatment, with cervical resection an option for fertility preservation. Adjuvant therapies such as pelvic radiation are often used post-surgery, while high-risk cases may require chemotherapy (3, 4). Stage II treatment involves radical resection of the cervix, uterus, and lymph nodes, supplemented with adjuvant therapies (3). For locally advanced (stage III) or metastatic (IVA-stage) disease, cisplatin-based chemotherapy remains the mainstay, with emerging immunotherapies like pembrolizumab showing promise in recurrent cases (5, 6). However, the clinical benefits of immunotherapy are inconsistent, with response rates below 20% in advanced CESC (7). This variability underscores the urgent need for predictive biomarkers to stratify patients likely to benefit from immunomodulatory therapies. A critical gap persists in understanding how immune-related biomarkers interact with the tumor microenvironment (TME) to influence prognosis. While studies have identified immune-targeted genes in CESC using gene microarrays (8), these efforts often focus on single biomarkers or lack integration with clinical outcomes (9). For instance, PD-L1 expression alone fails to predict immunotherapy response in 40–60% of CESC cases, suggesting that multi-gene signatures or immune contexture may better capture prognostic complexity (10). Furthermore, existing prognostic models rarely account for dynamic immune-TME crosstalk, limiting their utility in guiding personalized therapy (11).

To address these gaps, we propose a systems biology approach integrating multi-omics data and machine learning. By analyzing RNA-seq data from GEO and TCGA cohorts, we aim to identify immune-related gene signatures that not only predict survival but also reflect TME modulation. Our study diverges from prior work by prioritizing genes with dual roles in prognosis and immune regulation, validating biomarkers across heterogeneous cohorts, and constructing a nomogram that bridges molecular insights with clinical parameters (e.g., lymph node metastasis, tumor stage). This strategy addresses the limitations of reductionist biomarker discovery and provides a framework for translating immune-genomic findings into actionable clinical tools.

## 2 Materials and methods

### 2.1 Data collection and preprocessing

**Figure 1:**
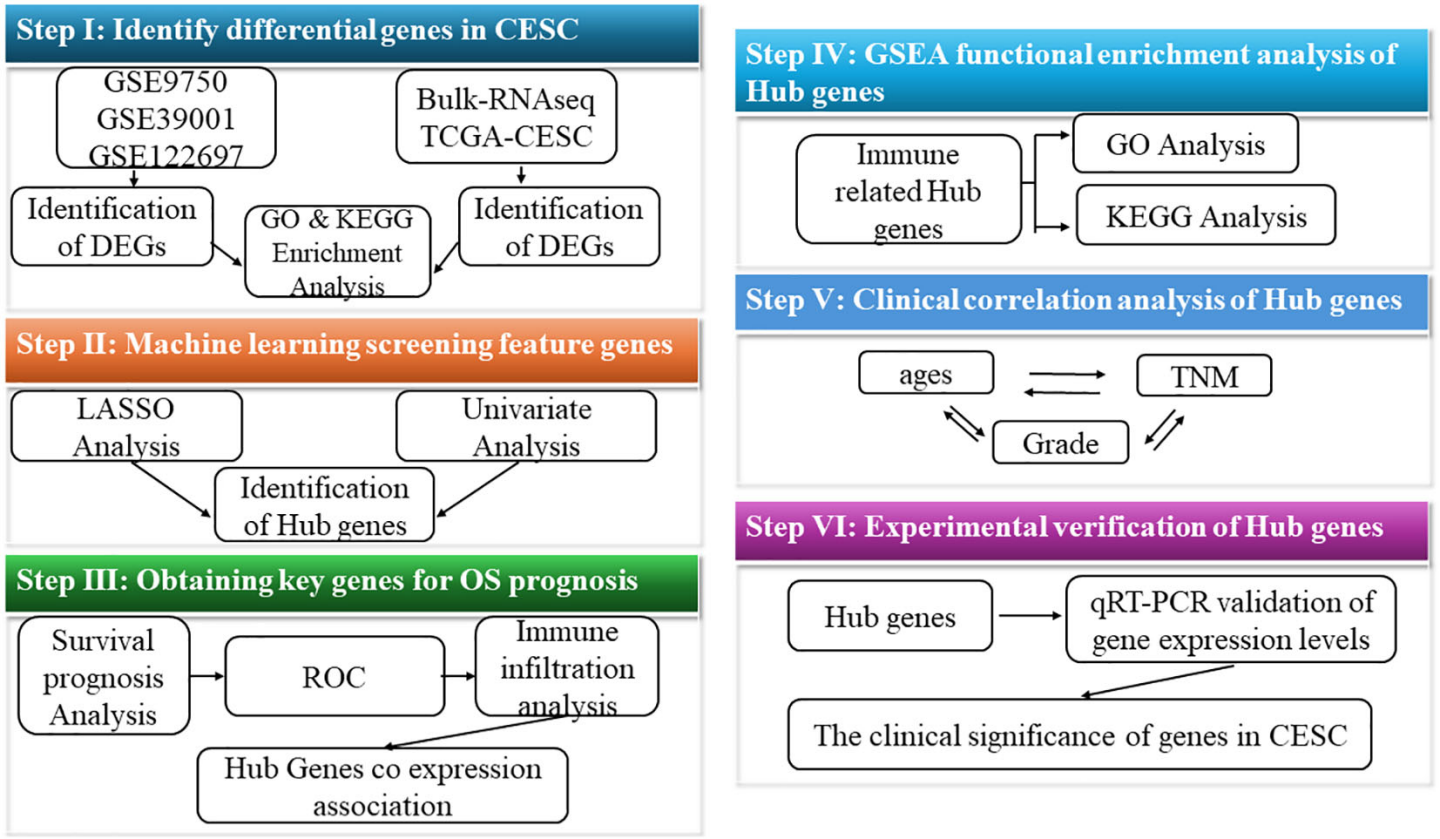
Research and design flow chart.

To better understand the research process, **Figure 1** illustrates the workflow diagram of this study. RNA-seq data were acquired from publicly accessible Gene Expression Omnibus (GEO) and The Cancer Genome Atlas (TCGA) databases (12, 13). The TCGA dataset contains clinical information from 296 cancer patients, including disease stage and survival outcomes, along with 3 normal tissue controls. Key characteristics of both datasets are systematically summarized in **Table 1**. DEGs were rigorously identified using the “limma” package in R, with selection criteria of |log2 FC|>1 and adjusted *P*<0.05 *(* 14).

**Table 1:** Details of GEO and TCGA dataset.

Dataset	Normal	Tumor
GSE9750	26	33
GSE39001	5	19
GSE122697	5	11
TCGA-ECSC	3	296

### 2.2 Functional enrichment analysis of GO and KEGG

DEGs were subjected to functional enrichment analysis using the DAVID database (https://david.ncifcrf.gov/) for Gene Ontology (GO) and Kyoto Encyclopedia of Genes and Genomes (KEGG) annotations (15). The “clusterProfiler” package in R facilitated statistical evaluation and visualization of enriched biological terms (16, 17). GO enrichment analysis includes three key biological domains: biological processes (BP), cellular components (CC), and molecular functions (MF) (18), while KEGG pathway annotation elucidated predominant metabolic and signaling pathways. Enrichment significance was determined at *P*<0.05.

### 2.3 Machine learning methods for obtaining disease characteristic genes

Feature selection employed LASSO regression and Support Vector Machine-Recursive Feature Elimination (SVM-RFE) (19, 20). The LASSO algorithm (“glmnet” package) identified CESC-associated genes through penalized regression (α=1), with optimal regularization parameter λ determined by five-fold cross-validation. SVM-RFE (“e1071” package) iteratively eliminated low-contribution features to extract disease-specific signatures. Prognostically significant differentially expressed genes were subsequently screened via univariate Cox regression (*P*<0.01), followed by multivariate Cox regression to construct the final predictive nomogram (21).

### 2.4 Validation of prognostic models

The risk score, derived from the constructed prognostic model, represents the prognostic risk for each CESC patient. The calculation formula for the model’s gene risk score is as follows: gene a coefficient multiplied by gene an expression, plus gene b coefficient multiplied by gene b expression,…, plus gene i coefficient multiplied by gene i expression (where a, b, and i represent specific genes). Training and testing cohorts were stratified into high-/low-risk groups using cohort-specific median thresholds. Survival disparities between risk strata were statistically validated through Kaplan-Meier analysis.

### 2.5 Estimation and analysis of immune cell infiltration patterns

Immune cell infiltration levels of 22 distinct subtypes were quantified through transcriptomic deconvolution using the “CIBERSORT” algorithm implemented in R. Comparative analysis of immune profiles between high- and low-risk cohorts was subsequently performed with the “limma” package.

### 2.6 GSEA enrichment analysis

Gene set enrichment analysis was conducted using the Molecular Signatures Database (MSigDB) collections “c5.go.v7.4.symbols” and “c2.cp.kegg.v7.4.symbols”. Hub genes associated with specific immune cell populations were functionally annotated through the “clusterProfiler” and “enrichplot” packages in R, with statistically significant terms identified at *P*<0.05 *(* 16).

### 2.7 Clinical general information

This retrospective analysis included 6 hysterectomy patients stratified into two cohorts: cervical squamous carcinoma (CESC; *n*=3, mean age 57.02 ± 9.21 years) and benign gynecological conditions (adenomyosis/uterine fibroids/prolapse; *n*=3, 57.56 ± 9.81 years), all confirmed by histopathological examination. Surgical specimens were snap-frozen in liquid nitrogen within 30 min post-resection for RNA preservation. Exclusion criteria encompassed pre-existing metabolic disorders (diabetes mellitus) or renal dysfunction. The research protocol was approved by the Second Affiliated Hospital of Guangdong Medical University (Approval No. PJKT2024-134).

### 2.8 RNA extraction and gene expression analysis

Gene expression analysis was performed using TRIzol reagent (Invitrogen, USA) to extract 1 μg total RNA per manufacturer’s protocol. RNA was reverse-transcribed into cDNA and amplified via qRT-PCR under standardized cycling conditions: 95°C for 5 min, followed by 40 cycles of 95°C for 15 s and 60°C for 30 s. Primer sequences used for qPCR are detailed in **Table 2**. Gene expression levels were quantified using the 2^–ΔΔCT^ method and normalized with *GAPDH*.

**Table 2:** Primer sequences for real-time reverse transcription PCR (qRT-PCR).

Gene name	Primer sequence (5′ to 3′)
*EFNA1* -Forward	CAGCGCTTCACACCTTTCAC
*EFNA1*-Reverse	GGTGGATGGGTTTGGAGATGT
*CXCL8*-Forward	AGCTCTGTGTGAAGGTGCAG
*CXCL8*-Reverse	TCTCAGCCCTCTTCAAAAACTTC
*PPP1R14A* -Forword	CACCGTCAAGTATGACCGGC
*PPP1R14A* -Reverse	GACTTCAGGAGTCCCATGCC
*GAPDH*-Forward	GGACTCATGACCACAGTCCAT
*GAPDH*-Reverse	CAGGGATGATGTTCTGGAGAG

### 2.9 Construction and validation of nomograms

A prognostic nomogram integrating independent risk factors identified through univariate and multivariate Cox regression analyses was developed using the rms package in R, enabling visualization of 3-, 5-, and 8-year overall survival (OS) predictions for CESC patients. Model validation comprised temporal discrimination assessment via time-dependent receiver operating characteristic (ROC) curves with area under the curve (AUC) quantification, calibration curve analysis for prediction accuracy evaluation.

### 2.10 Clinical relevance

Clinical utility of the nomogram was systematically evaluated through decision curve analysis (DCA). Using the ROC curve, we determine the optimal threshold for each patient’s risk score via the nomogram. Subsequently, patients in the training and validation queues are classified into high-risk and low-risk categories based on their calculated risk scores. Kaplan-Meier survival curves are employed to analyze OS between these groups in two cohorts to assess survival differences.

### 2.11 Statistical analysis

Statistical analyses were conducted using R software (version 4.3.1). Continuous and categorical variables were compared between groups using Student’s t-tests and Pearson’s chi-square tests, respectively. Survival outcomes were evaluated through Kaplan-Meier methodology with log-rank testing for group comparisons, supplemented by Cox proportional hazards regression modeling. Prognostic predictors were identified through univariate/multivariate Cox regression. Model discriminative ability was quantified using C-index, while calibration curves assessed prediction-observation agreement. Clinical decision-making utility was evaluated through DCA. Statistical significance in this study was determined using *P*<0.05.

## 3 Results

### 3.1 Screening for DEGs

**Figure 2:**
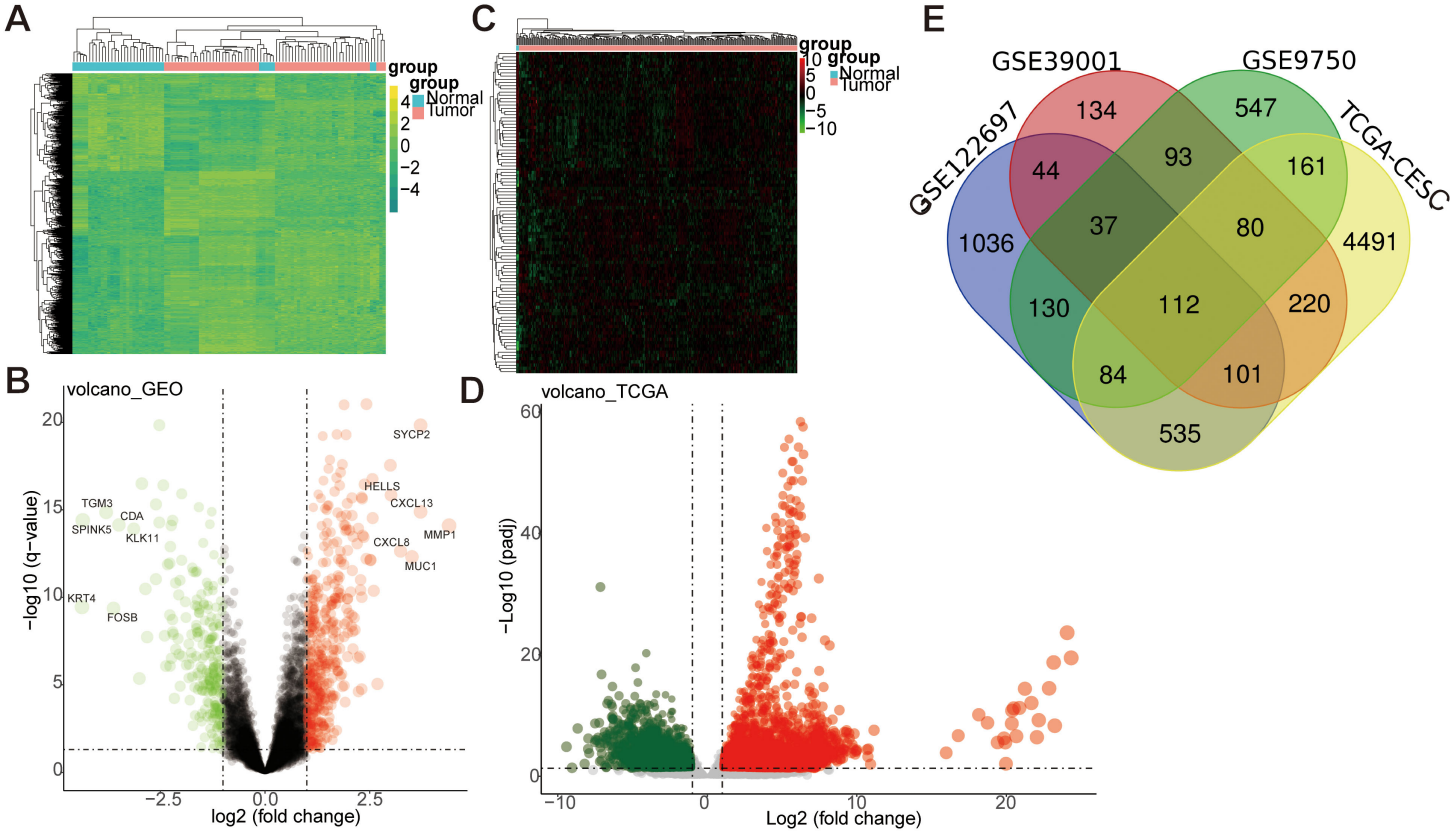
Differential gene analysis between GEO and TCGA datasets. **(A, B)** The differential gene analysis heatmap and volcano map of the GEO dataset. The expression profiles above the average in the heatmap are yellow, while those below the average are in green. **(C, D)** The differential gene analysis heatmap and volcano map of the TCGA dataset. Expression profiles above the average were represented in red. In contrast, those below the average were represented in green. Log FC: log2 fold changes. **(E)** Venn map of differentially expressed genes between GEO and TCGA datasets.

We retrieved mRNA expression profiles from GEO databases (GSE9750, GSE39001, GSE122697) to identify DEGs in CESC. Following batch calibration and normalization, differential expression analysis of the GEO dataset revealed 666 DEGs, comprising 434 upregulated genes (log2 FC>1) and 232 downregulated genes (log2 FC< -1), as shown in (**Figures 2A, B**). To further explore DEGs in CESC, we performed a detailed differential expression analysis using the TCGA dataset, comparing cancer tissues with adjacent normal tissues. This analysis identified 5,784 significantly differentially expressed genes, including 3,452 upregulated genes (log2 FC>1) and 2,332 downregulated genes (log2 FC<-1), as presented in (**Figures 2C, D**). By integrating the GEO and TCGA datasets, we identified 112 overlapping DEGs, consisting of 20 upregulated genes (log2 FC> 1) and 92 downregulated genes (log2 FC< -1), illustrated in (**Figure 2E**). This study conducted integrative differential gene expression analysis across GEO and TCGA cohorts, characterizing tumor-specific transcriptional dysregulation through systematic comparison of neoplastic and normal tissues.

### 3.2 GO & KEGG enrichment analysis of DEGs

**Figure 3:**
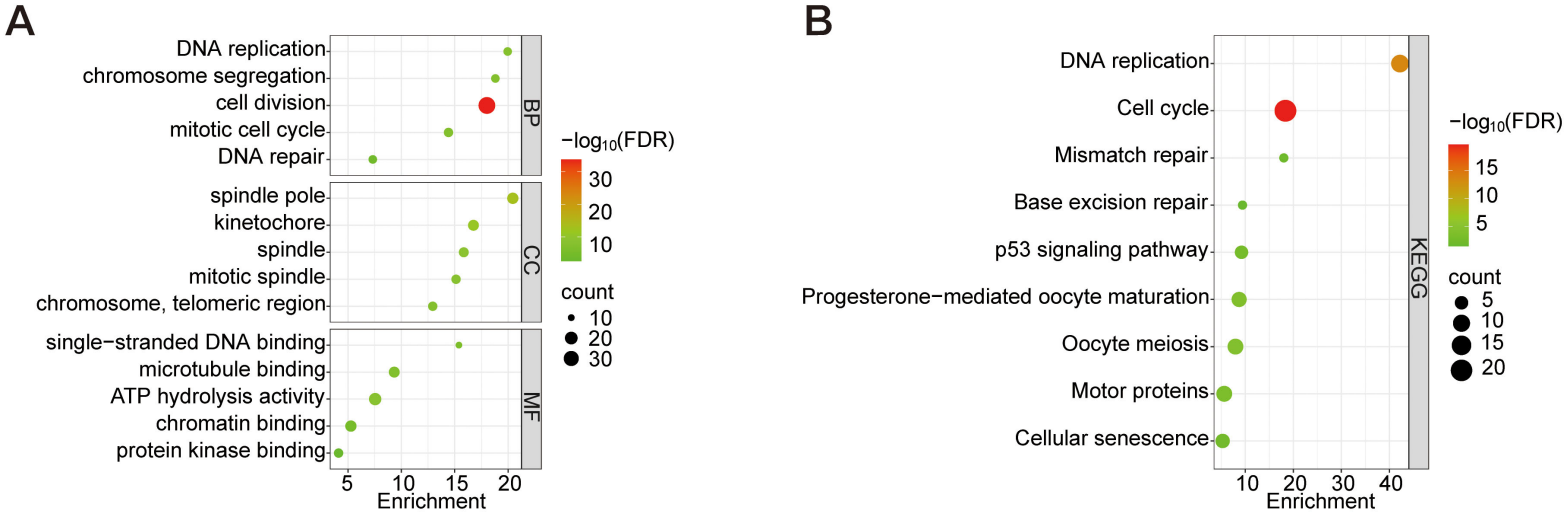
GO analysis of the top 5 enriched DEGs and KEGG analysis of the top 10 enriched DEGs. **(A)** GO enrichment analysis of BP, CC, and MF. **(B)** KEGG enrichment analysis. Node size represents the proportion of genes; The node color represents the *FDR* value.

To investigate the functional characteristics of 112 DEGs in CESC, systematic enrichment analyses were performed through the DAVID database. GO and KEGG analyses were conducted with *FDR*<0.05. Expressly, BP analysis indicated significant involvement of these DEGs in processes such as DNA replication, chromosome segregation, cell division, and the mitotic cell cycle. CC analysis revealed their primary association with spindle poles, kinetochores, and chromosome regions. Furthermore, MF analysis demonstrated that these DEGs primarily exhibit binding abilities to single-stranded DNA, microtubules, and protein kinases (**Figure 3A**). KEGG pathway analysis (**Figure 3B**) identified core mechanisms including Cell cycle, DNA replication, Oocyte meiosis, Progesterone-mediated oocyte maturation, Motor proteins, p53 signaling pathway, Cellular senescence, Mismatch repair, and Base excision repair. These findings collectively implicate the DEGs in essential oncogenic processes: genomic stability maintenance through DNA replication/repair, mitotic regulation via spindle apparatus components, and potential involvement in cellular senescence mechanisms in CESC pathogenesis.

### 3.3 LASSO regression of differentially expressed genes in GEO dataset and SVM-RFE algorithm for screening key pathogenic-characteristic gene

**Figure 4:**
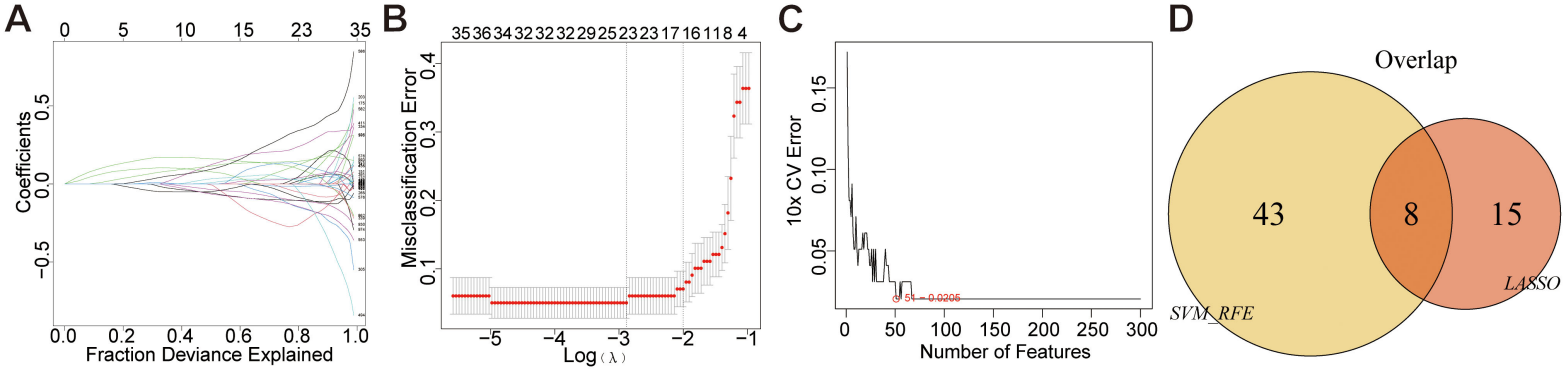
Mechanical learning method for obtaining disease characteristic genes from GEO dataset. **(A, B)** LASSO regression plot, λ = 0.1022. **(C)** SVM-RFE diagram, 51-0.0205 indicates an error rate of 0.0205 for the 51 trait genes screened. **(D)** Venn diagram.

Disease-associated genes were identified through a multi-step feature selection process. LASSO regression analysis selected 23 characteristic genes (e.g., *CXCL8*, *EFNA1*, *EZH2*) by minimizing prediction error, with error magnitude and gene number relationships visualized (**Figures 4A, B**). SVM-RFE analysis subsequently refined candidate genes through recursive elimination, identifying 51 optimal features at minimal cross-validation error (**Figure 4C**). Intersection analysis of both methods revealed eight core disease-characteristic genes (*CXCL8*, *EFNA1*, *EZH2*, *PAQR4*, *SLC27A6*, *SPINK5*, *SYCP2*, *YEATS2*), confirmed by Venn diagram (**Figure 4D**).

### 3.4 Screening of disease characteristic genes using LASSO regression and univariate COX regression analysis in the TCGA dataset

**Figure 5:**
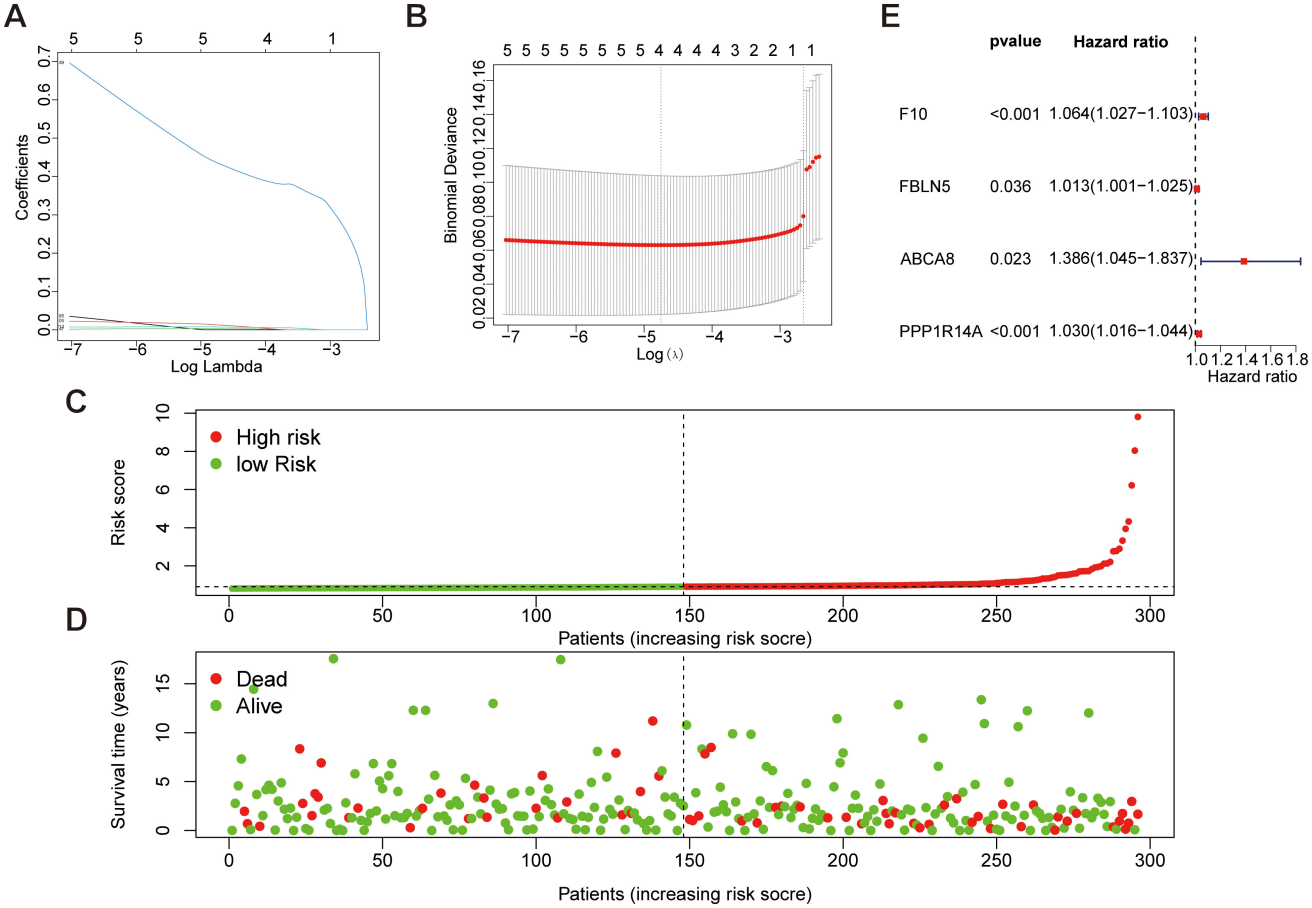
Mechanical learning method for obtaining disease characteristic genes from TCGA dataset. **(A, B)** LASSO regression plot, λ=0.0086. **(C, D)** Patient risk scores and survival status for high-risk and low-risk groups. **(E)** Forest plot of key genes selected in features through univariate Cox analysis.

Feature selection was conducted through LASSO regression (“glmnet” package) to identify feature genes demonstrating minimal prediction error. The optimal regularization parameter (λ) was determined via cross-validation, selecting hub genes with the lowest error rate as disease-specific biomarkers (**Figures 5A, B**). Furthermore, a risk-scoring model for cervical cancer was constructed and used to stratify 296 CESC patients into low-risk (*n*=148) and high-risk (*n*=148) groups based on their median disease risk, as shown in (**Figure 5C**). For further insight, (**Figure 5D**) displays the risk score distribution and corresponding survival status of patients within these risk groups. Finally, univariate Cox regression analysis was conducted to identify four key genes (*F10*, *PPP1R14A*, *FBLN5*, *ABCA8*), and a gene model was constructed based on these findings. The analysis results are presented clearly in (**Figure 5E**).

### 3.5 Survival prognosis analysis of key genes

**Figure 6:**
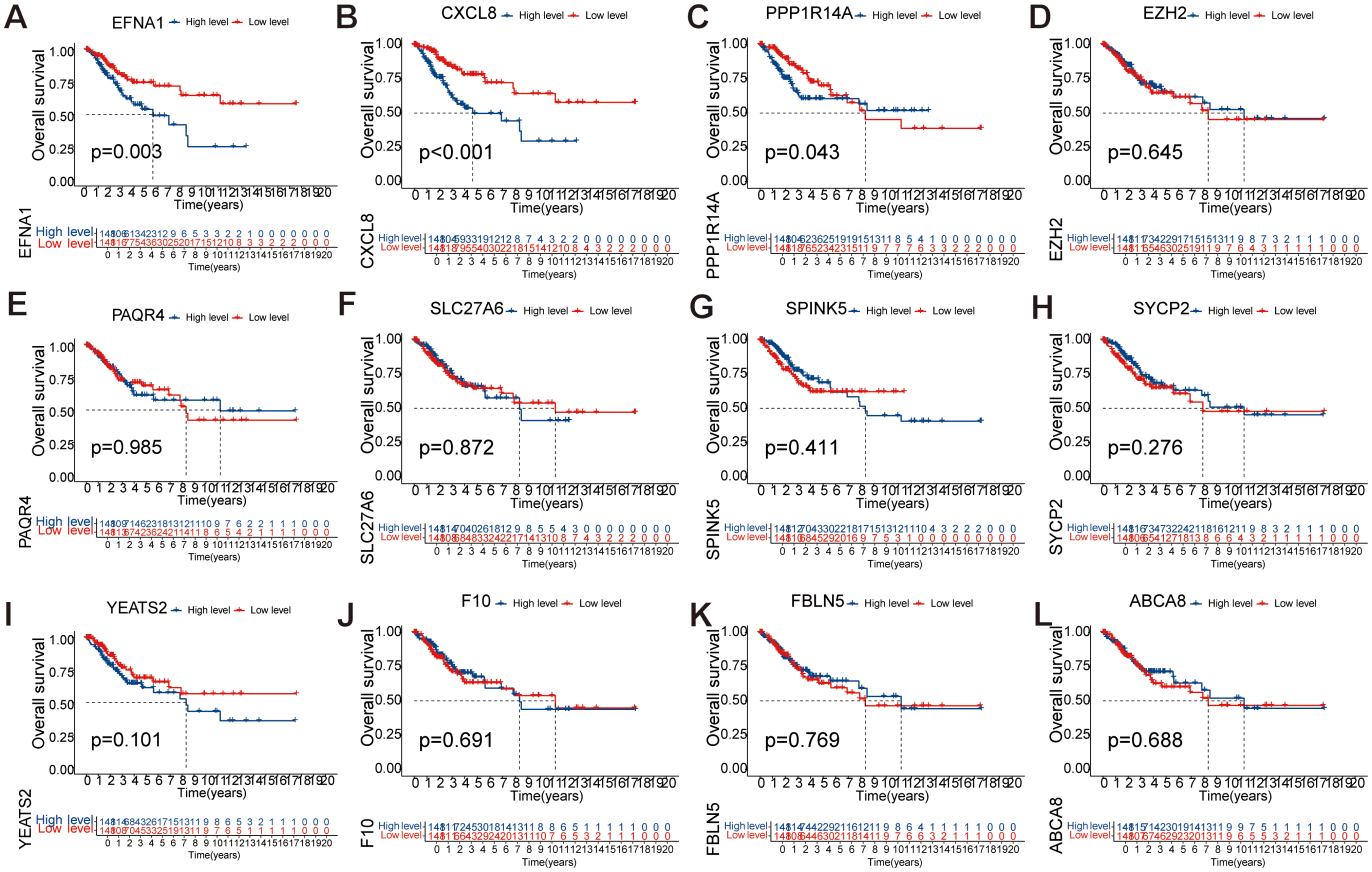
Single-factor analysis of factors influencing the survival rates of CESC patients. **(A-L)** Kaplan Meier survival curve analysis of *EFNA1*, *CXCL8*, *EZH2*, *PAQR4*, *SLC27A6*, *SPINK5*, *SYCP2*, *YEATS2*, *F10*, *PPP1R14A*, *FBLN5*, and *ABCA8* showed significant differences in OS between low-risk and high-risk scoring groups.

To investigate the prognostic significance of 12 key genes (*EFNA1*, *CXCL8*, *EZH2*, *PAQR4*, *SLC27A6*, *SPINK5*, *SYCP2*, *YEATS2*, *F10*, *PPP1R14A*, *FBLN5*, *ABCA8*) in CESC, we conducted a comprehensive survival analysis. Notably, *EFNA1* (**Figure 6A**), *CXCL8* (**Figure 6B**), and *PPP1R14A* (**Figure 6C**) demonstrated statistically significant predictive value for overall survival (*P<*0.05). Conversely, *EZH2*, *PAQR4*, *SLC27A6*, *SPINK5*, *SYCP2*, *YEATS2*, *F10*, *FBLN5*, and *ABCA8* did not exhibit statistically significant differences in overall survival prognosis (**Figures 6D–L**). These findings suggest that *EFNA1*, *CXCL8*, and *PPP1R14A* may serve as potential prognostic markers for CESC, warranting further investigation for their roles in disease progression and therapeutic targeting.

### 3.6 ROC validation of key survival prognostic genes

**Figure 7:**
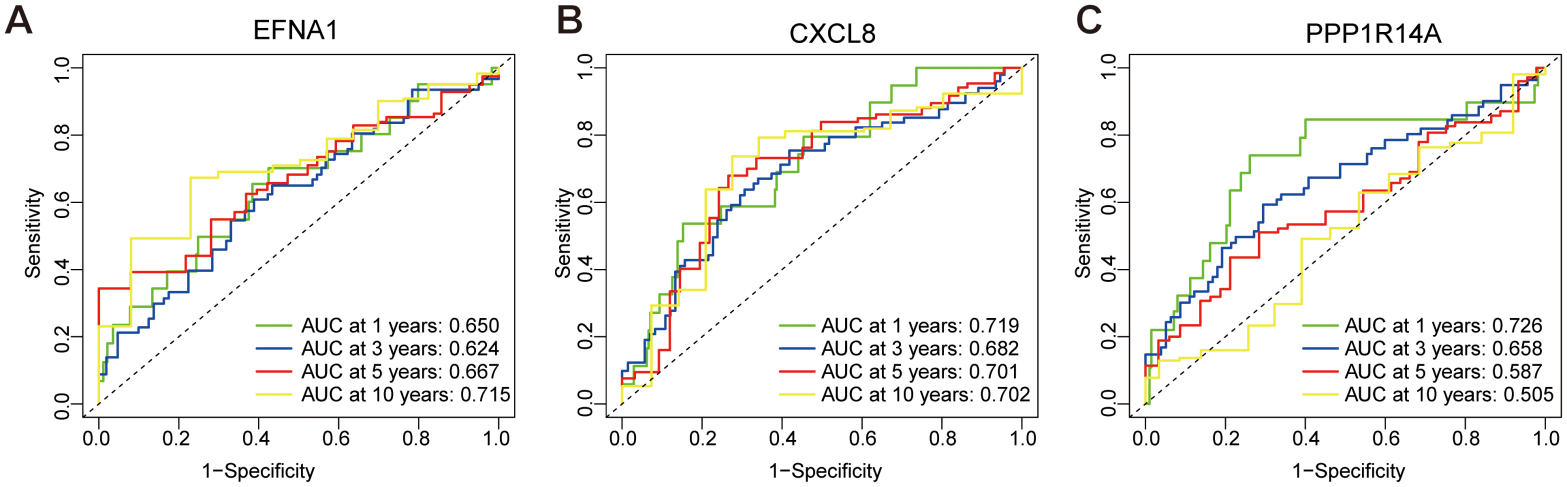
ROC curve for predicting overall survival based on risk score. **(A)** ROC analysis of *EFNA1* gene. **(B)** ROC analysis of *CXCL8* gene. **(C)** ROC analysis of *PPP1R14A* gene.

Time-dependent ROC analysis evaluated the prognostic accuracy of a three-gene signature (*EFNA1*, *CXCL8*, *PPP1R14A*) for cervical squamous cell carcinoma survival outcomes. The *EFNA1* (**Figure 7A**) demonstrated moderate discrimination with progressive improvement in AUC from 0.650 (1-year) to 0.715 (10-year), consistently exceeding random prediction. *CXCL8* (**Figure 7B**) showed superior and stable performance across all intervals, maintaining AUC between 0.682-0.719 with minimal temporal variation. Notably, *PPP1R14A* (**Figure 7C**) exhibited temporal performance heterogeneity - achieving peak discriminative capacity at 1-year (AUC=0.726) followed by progressive decline to 0.505 at 10-year follow-up. This temporal analysis revealed two distinct prognostic patterns: *CXCL8* maintained consistent predictive reliability throughout the observation period, while *PPP1R14A* and *EFNA1* showed opposing temporal trajectories. The composite model demonstrated the strongest predictive utility for early-stage prognosis (1–3 year AUC range: 0.650-0.726), with diminished accuracy in long-term predictions (5–10 year AUC range: 0.505-0.715). These findings position this multigene signature as a potential tool for short-term survival stratification, though longitudinal prognostic accuracy may require temporal recalibration or incorporation of complementary biomarkers.

### 3.7 Key gene immune infiltration analysis

**Figure 8:**
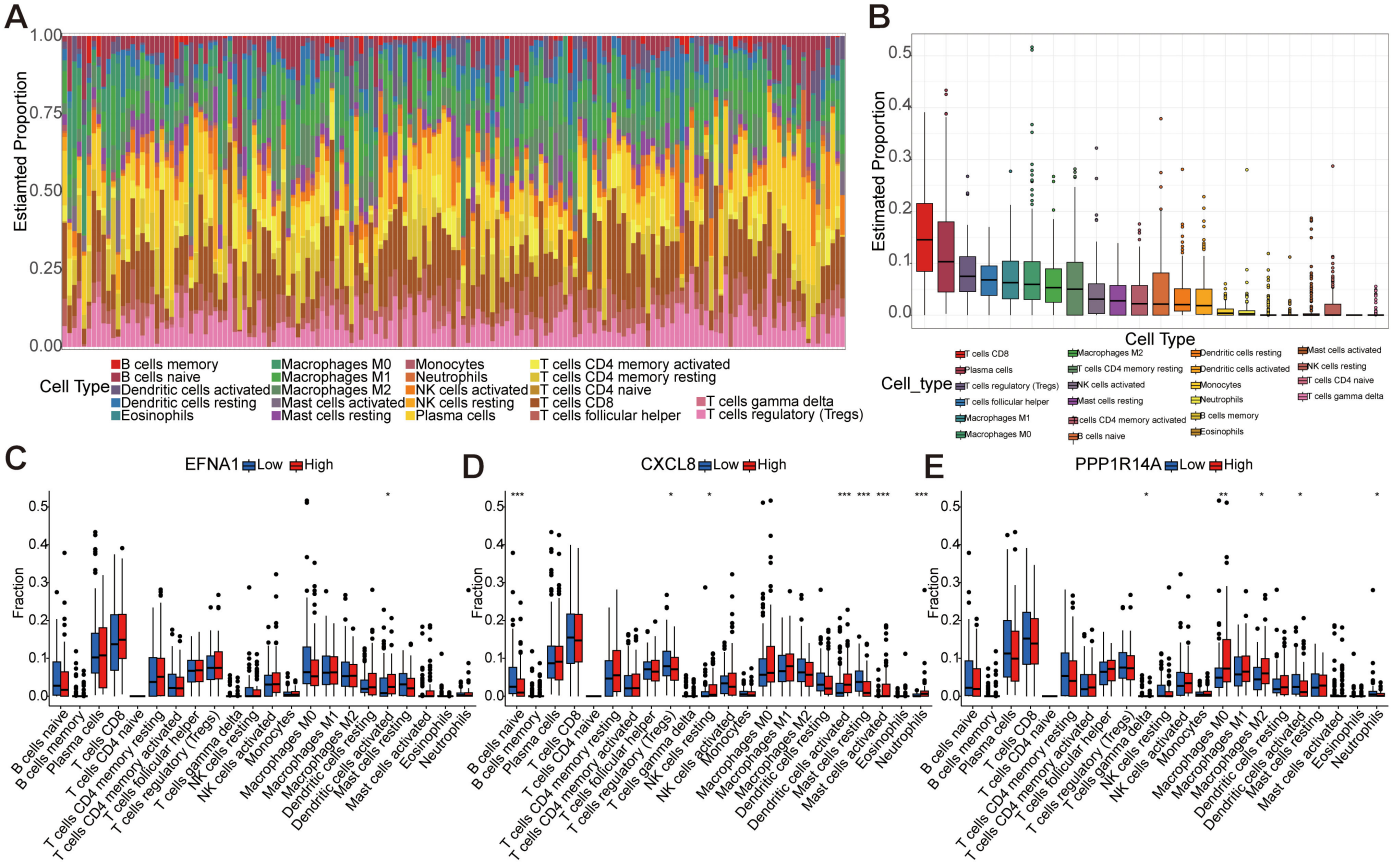
Immune infiltration analysis based on 22 immune-related gene sets. **(A)** Representing 22 subtypes of immune cells, the abundance of each bar chart represents the proportion of immune cells in each sample, and different colors represent each subtype. **(B)** Differences in the proportion of 22 subtypes of immune cells. **(C-E)** The comparative analysis of the proportion of immune infiltrating cells in the high-risk and low-risk groups of *EFNA1*, *CXCL8*, and *PPP1R14A* (*P<0.05, **P<0.01, ***P<0.001).

Comprehensive immune microenvironment profiling of CESC was performed through analysis of 22 immune cell signatures and infiltration patterns (**Figures 8A, B**). The heatmap visually represents the abundance and proportional differences of various immune cell subtypes in CESC samples, highlighting a higher proportion of CD8+ T cells, plasma cells, Tregs, follicular helper T cells, and macrophages (M1, M0, and M2). To explore the correlation between the three key genes (*EFNA1*, *CXCL8*, and *PPP1R14A*) and immune cell infiltration, we stratified the gene expression levels into high- and low-risk groups for analysis (**Figures 8C-E**). The findings revealed significant differences in dendritic cell activation between high- and low-risk groups for *EFNA1* (*P*<0.05). *CXCL8* significantly influenced the proportions of Tregs, NK cells, dendritic cell activation, mast cells, and neutrophils (*P*<0.05), and changes in *PPP1R14A* were significantly associated with γδ T cells, macrophages (M0 and M2), dendritic cell activation, and the proportion of neutrophils (*P*<0.05). These mechanistic insights propose clinically actionable strategies: *CXCL8* inhibition may disrupt immunosuppressive networks while *PPP1R14A* modulation could target macrophage polarization, with *EFNA1*-mediated dendritic cell activation serving as a potential predictive biomarker for immune checkpoint inhibitor response.

### 3.8 Specific gene co-expression analysis

**Figure 9:**
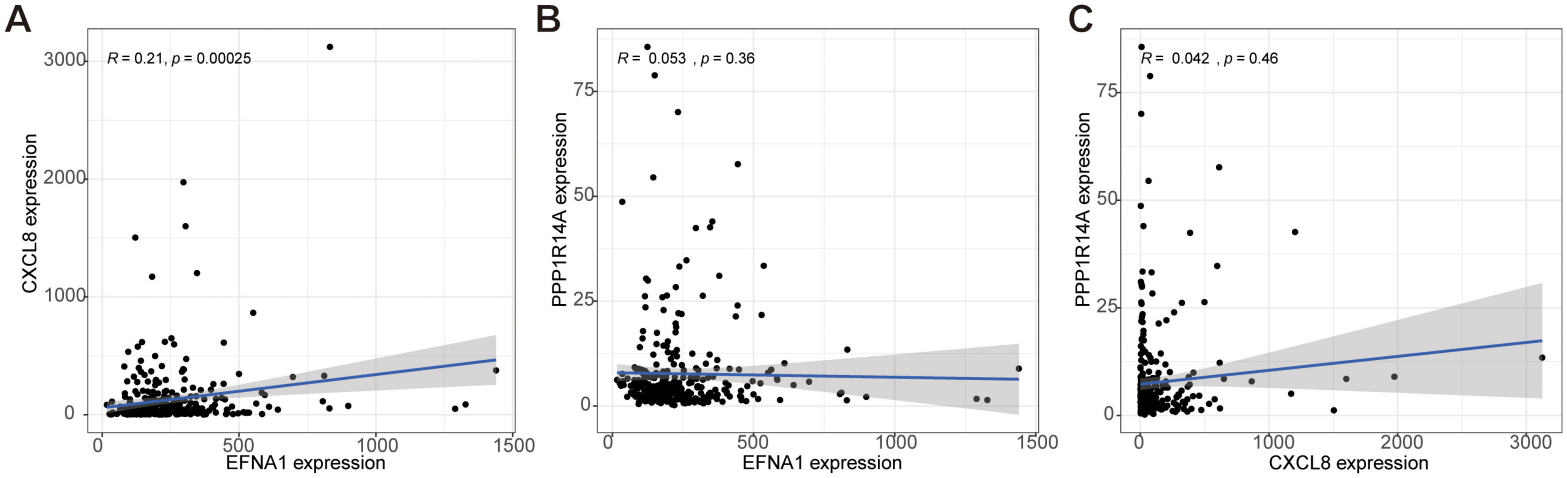
Analysis of the co-expression correlation for key genes *EFNA1*, *CXCL8*, and *PPP1R14A*. **(A–C)** The correlation among *EFNA1*, *CXCL8*, and *PPP1R14A* expressions in our cohort was determined by Spearman's correlation analysis.

Gene co-expression network analysis revealed distinct interaction patterns among *EFNA1*, *CXCL8*, and *PPP1R14A*. Pearson correlation analysis demonstrated a statistically robust positive correlation between *EFNA1* and *CXCL8* expression (R=0.21, *P*<0.001; **Figure 9A**), suggesting potential co-regulatory mechanisms or functional synergy. In contrast, non-significant correlations were observed for *EFNA1*-*PPP1R14A* (R=0.053, *P*=0.36; **Figure 9B**) and *CXCL8*-*PPP1R14A* pairs (R=0.042, *P*=0.46; **Figure 9C**), as evidenced by nullcline-proximal data distributions. This differential correlation profile implies *PPP1R14A*’s functional autonomy from the *EFNA1*-*CXCL8* axis within the studied biological context.

### 3.9 Enrichment analysis of key survival prognostic genes using GSEA

**Figure 10:**
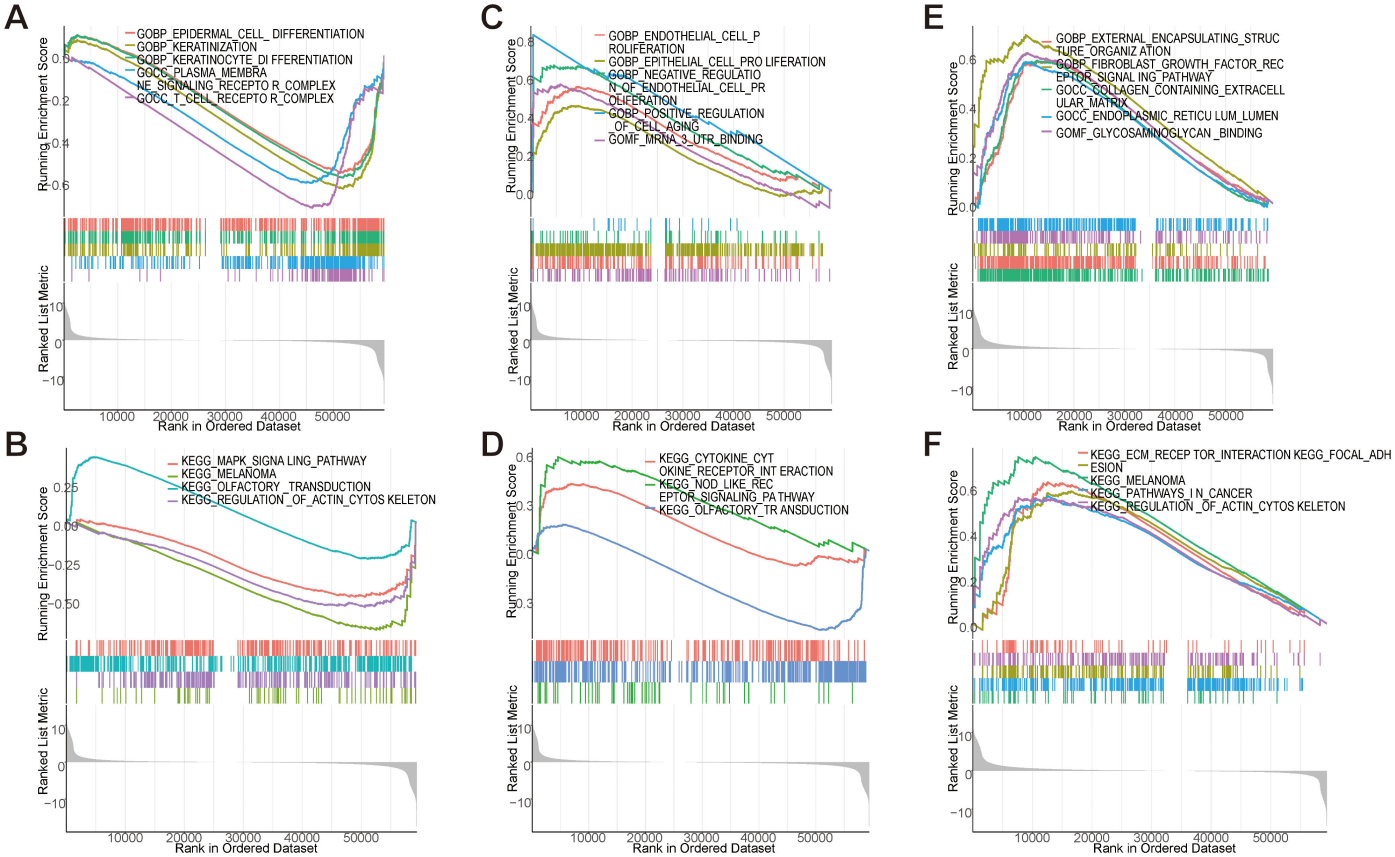
GSEA enrichment analysis of key genes. **(A, B)** GSEA analysis of *EFNA1* gene. **(C, D)** GSEA analysis of *CXCL8* gene. **(E, F)** GSEA analysis of *PPP1R14A* gene.

GSEA systematically decoded functional networks of *EFNA1*, *CXCL8*, and *PPP1R14A* within the CESC immune microenvironment. *EFNA1* exhibited dual regulatory capacity: GO terms implicated its involvement in keratinocyte differentiation and T cell receptor complex assembly (**Figure 10A**), while KEGG pathways connected it to MAPK signaling and cytoskeletal remodeling (**Figure 10B**). *CXCL8* exhibited dual regulatory roles in cervical carcinogenesis, demonstrating pro-proliferative effects on endothelial/epithelial lineages (**Figure 10C**) concurrent with inflammasome activation through NOD-like receptor signaling pathways (**Figure 10D**). *PPP1R14A* emerged as a stromal interface regulator, with GO enrichment in fibroblast growth factor signaling and extracellular matrix (ECM) organization (**Figure 10E**), corroborated by KEGG pathways encompassing ECM-receptor interactions and cancer-associated adhesion molecules (**Figure 10F**). Multi-dimensional analysis reveals three synergistic pathological mechanisms in cervical squamous carcinogenesis: *EFNA1*-mediated immune-structural interactions regulate differentiation processes, *CXCL8* coordinates proliferative-inflammatory equilibrium, and *PPP1R14A* drives stromal-ECM remodeling. These interconnected networks collectively promote tumor progression through differentiation dysregulation, immune evasion, and metastatic niche formation, identifying microenvironment-specific targets for precision immunotherapy development.

### 3.10 Clinical correlation analysis of key genes for survival prognosis

**Figure 11:**
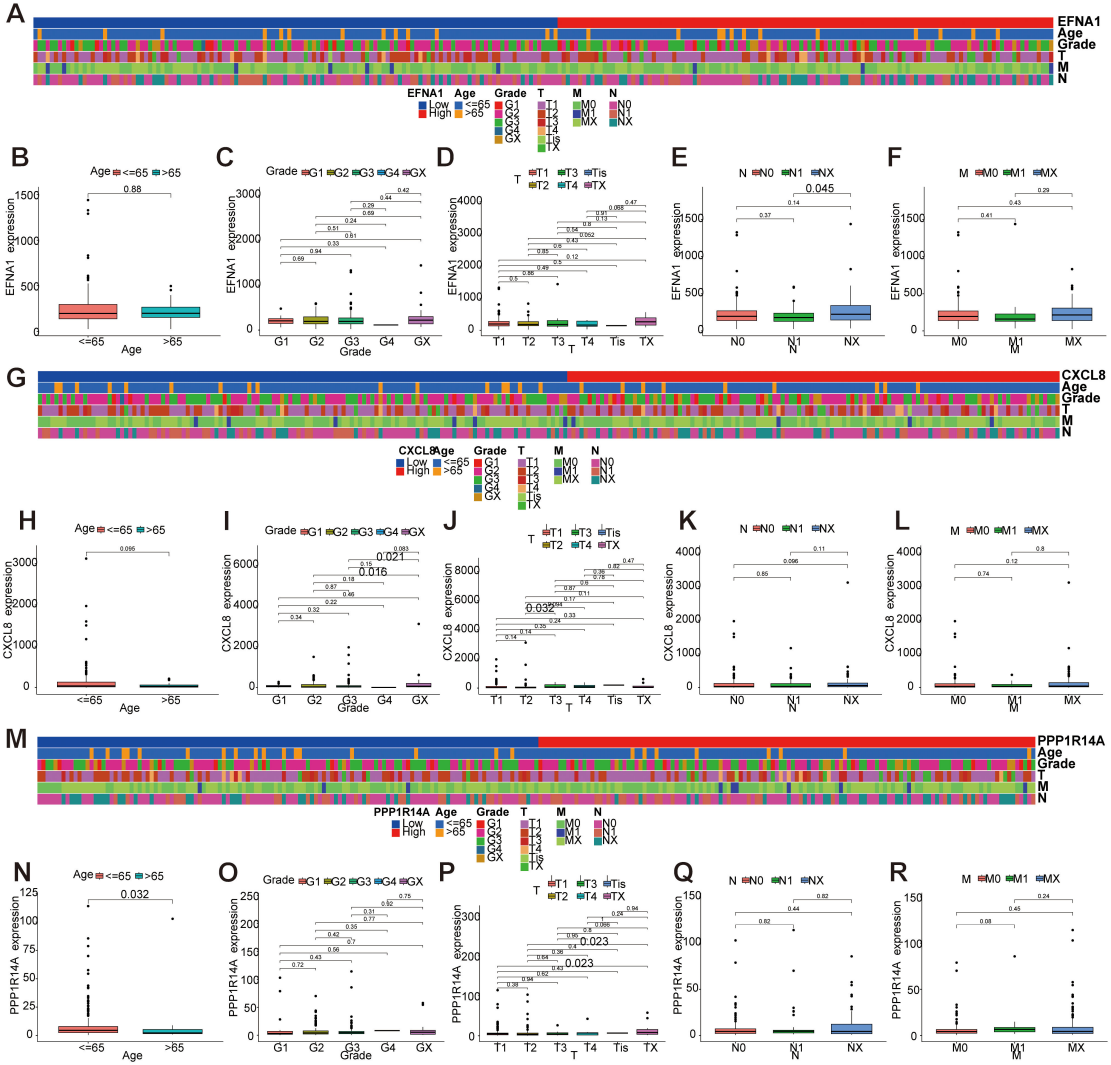
Correlation analysis between key gene expression and clinical features. **(A-F)** Correlation analysis between *EFNA1* gene expression and clinical features. **(G-L)** Correlation analysis between gene expression of *CXCL8* and clinical features. **(M-R)** Correlation analysis between *PPP1R14A* gene expression and clinical features.

To investigate the role of key genes in cervical cancer survival prognosis, we comprehensively analyzed the correlation between the expression levels of *EFNA1*, *CXCL8*, and *PPP1R14A*, and various clinical features, including age, stage, tumor grade (T), lymph node status (N), and metastasis status (M). *EFNA1* demonstrated lymph node-specific regulation with elevated expression in NX versus N1 cases (*P*=0.045, **Figures 11A, E**), showing no significant associations with age, stage, grade, or metastasis (**Figures 11B-D, F**). *CXCL8* exhibited progressive upregulation in advanced disease stages (T3 vs T2, *P*=0.032; G3 vs G2, P=0.016; **Figures 11G-J**), independent of age or metastatic status (**Figures 11K, L**). *PPP1R14A* displayed age-dependent suppression (>65 years, *P*=0.032) and paradoxical elevation in TX-grade tumors (vs T1-2, *P*=0.023; **Figures 11M, N, P**), with no significant correlations to staging or metastasis (**Figures 11O, Q, R**). In short, *EFNA1* demonstrates lymph node-specific biomarker potential, *CXCL8* correlates with histopathological progression through stage/grade associations, and *PPP1R14A* exhibits age-related suppression and grade-dependent paradoxical expression. These differential clinical signatures collectively highlight their translational value for precision staging systems and biomarker-driven therapeutic stratification in CESC management.

### 3.11 Establishment and evaluation of a prognostic nomogram for key genes and clinical features

**Figure 12:**
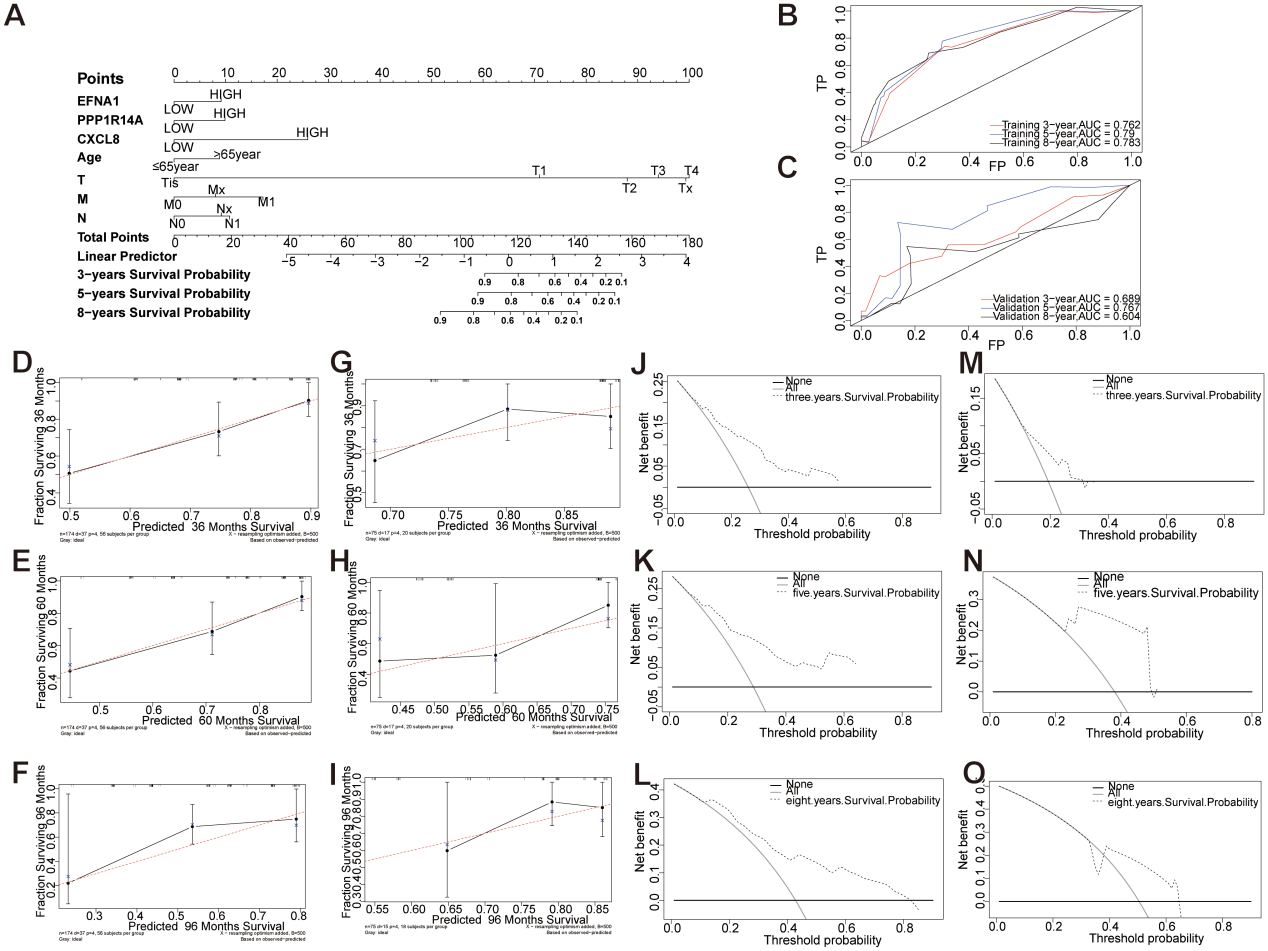
Establishment and evaluation of a prognostic nomogram of key genes and clinical features. **(A)** Models of OS for CESC patients at 3, 5, and 8 years. **(B, C)** Time-dependent curves (ROC) of the nomogram for 3-, 5-, and 8-year predictions in the training and validation cohorts. **(D-I)** Calibration charts of 3-year, 5-year, and 8-year overall survival for CESC patients in the training and validation cohorts. **(J-O)** Decision curve analysis of nomogram. DCA curves of 3-year, 5-year, and 8-year OS in the training queue and validation queue.

A prognostic nomogram integrating three molecular markers (*EFNA1*, *CXCL8*, *PPP1R14A*) with clinicopathological parameters (age, TNM stage) was developed to predict 3-/5-/8-year overall survival in cervical squamous cell carcinoma (**Figure 12A**). Tumor grade demonstrated the strongest prognostic weight (C-index: 0.785 training, 0.750 validation), followed by *CXCL8* expression and metastasis status. The nomogram showed robust temporal discrimination (3-year AUC: 0.762 training, 0.689 validation; **Figures 12B, C**) and precise calibration (**Figures 12D-I**), with DCA confirming clinical utility across 10-45% risk thresholds (**Figures 12J-O**). This multivariable tool enables personalized survival prediction and risk-stratified therapeutic decision-making for CESC patients.

### 3.12 Key gene qRT-PCR validation

**Figure 13:**
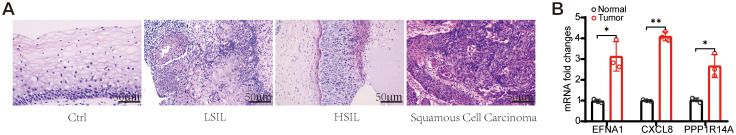
Morphological changes and Hub gene expression validation in CESC patients. **(A)** Typical HE staining images of CESC patients. (scale bar=50 μm). **(B)** *EFNA1*、*CXCL1* and *PPP1R14A* mRNA expression levels were examined using qRT-PCR. (*n*=3, *P<0.05, **P<0.01 vs Normal).

Cervical cancer primarily consists of squamous cell carcinoma (70%), adenocarcinoma (25%), and other rare types (5%) (22). Multimodal analysis of cervical carcinogenesis progression was conducted through histopathological evaluation and molecular profiling. Hematoxylin-eosin staining revealed progressive histoarchitectural alterations (**Figure 13A**): Control tissues maintained normal stratified squamous epithelium, LSIL specimens exhibited basal layer expansion with koilocytic changes, HSIL showed full-thickness dysplasia with nuclear hyperchromasia, and invasive carcinoma displayed complete architectural disarray with stromal invasion. Complementary qRT-PCR analysis demonstrated significant transcriptional upregulation of oncogenic effectors - *EFNA1*, *CXCL8*, and *PPP1R14A* in carcinoma versus control tissues (*P*<0.05) (**Figure 13B**). This coordinated overexpression pattern correlates with histopathological progression from premalignant lesions to invasive carcinoma, suggesting their synergistic role in cervical carcinogenesis.

## 4 Discussion

CESC poses a substantial threat to women’s health worldwide, with epidemiological studies showing ~80% of cases diagnosed at advanced stages (III-IV) where treatment efficacy remains limited (23, 24). Although therapeutic strategies have evolved, patients with advanced CESC still demonstrate poor clinical outcomes, emphasizing the imperative need for both validated prognostic biomarkers and elucidation of molecular progression mechanisms. In this study, we identified *EFNA1*, *CXCL8*, and *PPP1R14A* as core biomarkers that bridge prognosis with immune modulation, offering novel insights into CESC biology and therapeutic targeting.

*EFNA1*, a member of the Ephrin ligand family, regulates cell migration and angiogenesis through Eph receptor interactions (25, 26). Studies have shown that *EFNA1* was associated with MAPK signaling, a pathway driving proliferation and invasion in CESC (27). In CESC, *EFNA1*’s association with lymph node metastasis (*P*=0.045) and may drive invasiveness via Eph receptor-mediated MAPK/STAT3 activation, a pathway linked to epithelial-mesenchymal transition (EMT) in solid tumors. *CXCL8*, a pro-inflammatory chemokine, was linked to NOD-like receptor signaling, which activates inflammasomes and modulates immune responses (28). In CESC, *CXCL8* fosters an immunosuppressive tumor microenvironment by recruiting neutrophils and regulatory T cells (Tregs) (29), consistent with its correlation to advanced tumor stages and immune cell infiltration patterns. *PPP1R14A*, a member of the protein phosphatase 1 (*PP1*) inhibitor family, is also known as the 17kDa PKC-enhanced *PP1* inhibitory protein (*CPI-17*) (30). Studies have shown that *PPP1R14A* plays a crucial role in the onset and progression of various tumors, including sporadic vestibular glioma, human melanoma, and schwannoma (30–32). *PPP1R14A* was associated with fibroblast growth factor signaling and extracellular matrix (ECM) organization, critical for stromal remodeling and tumor invasion (33). Its clinical correlations with tumor grades suggest it contributes to invasive phenotypes through ECM alterations. These pathways suggest that *EFNA1*, *CXCL8*, and *PPP1R14A* drive CESC progression through proliferation, immune modulation, and stromal alterations, respectively. Their interconnected roles merit further exploration.

The immune infiltration analysis further contextualized our findings by revealing elevated infiltration of CD8^+^ T cells, Tregs, and tumor-associated macrophages (TAMs) in CESC specimens. The activation of *EFNA1* and mast cells provides a new target for Ephrin signaling in allergic diseases, highlighting tissue-specific immune modulation (34). *EFNA1* correlated with dendritic cell activation (*P*<0.05), potentially enhancing antigen presentation. *CXCL8* was associated with increased Tregs, NK cells, and neutrophils (*P*<0.05), supporting its immunosuppressive role (29). *PPP1R14A* influenced γδ T cells and M2 macrophages (*P*<0.05), linked to tumor progression (35, 36). However, this study did not assess survival outcomes tied to immune cell abundance—a limitation, as high Treg infiltration often predicts poor prognosis in cancers (37). Future survival analyses are needed to clarify these associations in CESC.

A prognostic nomogram integrating *EFNA1*, *CXCL8*, *PPP1R14A*, age, and TNM stage achieved strong predictive accuracy for 3-, 5-, and 8-year survival (3-year AUC: 0.762–0.763), with decision curve analysis confirming its utility. Recent studies have shown that *EFNA1* overexpression serves as an independent prognostic risk factor in cervical cancer, demonstrating robust predictive value for survival outcomes (25, 38). CXCL8 plays a role in modulating immune infiltration, thereby influencing the prognosis of patients with various cancers, particularly cervical cancer (39–41). While research on *PPP1R14A* in CESC is limited, studies have shown that its high expression in bladder cancer (BCA) is associated with poor prognosis, suggesting its potential as a prognostic biomarker (42). Unlike prior models focusing on single biomarkers (43, 44), our nomogram combines multi-gene signatures with clinical parameters, achieving superior predictive accuracy. Experimental validation via qRT-PCR and HE staining showed significant upregulation of all three genes in CESC tissues (*P*<0.05), along with histopathological progression from normal epithelium to invasive carcinoma, further supporting their biological relevance.

However, the retrospective nature of TCGA and GEO data may introduce selection bias. Computational findings regarding immune infiltration require confirmation through immunohistochemistry or flow cytometry. Future research should validate these biomarkers in prospective cohorts and explore their functional roles through single-cell sequencing or knockout models. Additionally, prospective validation of the nomogram and survival analyses examining the relationship between immune cell abundance and clinical outcomes are crucial next steps.

## 5 Conclusion

In this study, we developed a predictive risk model for CESC by integrating *EFNA1*, *CXCL8*, and *PPP1R14A* gene expression profiles with clinical baseline characteristics. This prognostic nomogram demonstrates strong predictive power while requiring only a minimal gene panel, thereby reducing economic burdens on patients and showing potential for clinical application and translational research. Furthermore, the nomogram independently predicts patient prognosis and complements existing TNM staging systems, offering comprehensive information to support clinical decision-making. With future validation in more clinical cases, our research is poised to benefit a broader patient population and propel advancements in personalized cancer treatment and precision medicine.

## Data Availability

Publicly available datasets were analyzed in this study. This data can be found here: Publicly available datasets were analyzed in this study. This data can be found at: TCGA (https://portal.gdc.cancer.gov/); GEO (https://www.ncbi.nlm.nih.gov/geo/) with accession numbers: GSE9750、GSE39001 and GSE122697.
